# Outcomes of Intracardiac Echocardiography as the Primary Imaging Modality for Transcatheter Tricuspid Valve Procedures

**DOI:** 10.1016/j.shj.2025.100662

**Published:** 2025-05-21

**Authors:** Anas I. Zaqut, Ashtyn Chamberland, Scott M. Chadderdon, Firas E. Zahr

**Affiliations:** Division of Cardiovascular Medicine, Oregon Health and Science University, Portland, Oregon, USA

**Keywords:** 3D ICE, 3D TEE, Multiplanar reconstruction, T-TEER, TTVR

## Abstract

**Background:**

Tricuspid regurgitation (TR) is a high-mortality condition. While tricuspid transcatheter edge-to-edge repair (T-TEER) and transcatheter tricuspid valve replacement (TTVR) improve quality of life, their reliance on transesophageal echocardiography (TEE) poses challenges. Intracardiac echocardiography (ICE) offers a promising adjunct; however, data on safety and efficacy remain limited. This study evaluates procedural characteristics, safety, and outcomes as operators gain experience with ICE for tricuspid procedures.

**Methods:**

Twenty-two patients with symptomatic ≥ severe TR treated with TTVR or T-TEER at Oregon Health & Science University were enrolled. All procedures utilized ICE and TEE. Patients were divided into 2 cohorts: cohort 1 primarily used TEE with adjunctive ICE (10 cases: 4 TTVRs and 6 T-TEERs), while cohort 2 primarily used ICE with adjunctive TEE (12 cases: 5 TTVRs and 7 T-TEERs). Procedural characteristics and outcomes were compared.

**Results:**

Mean TTVR procedure time decreased from 124 ± 38 minutes in cohort 1 to 98 ± 36 minutes in cohort 2, while T-TEER times dropped from 115 ± 35 minutes to 108 ± 30 minutes. Fluoroscopy time decreased from 27 ± 10 minutes to 19 ± 9 minutes for TTVRs and from 27 ± 11 minutes to 25 ± 10 minutes for T-TEERs. The TR grade was reduced by ≥ 2 grades in all but 1 patient with no mortality, bleeding events, or hospitalizations across all cohorts.

**Conclusions:**

Primary ICE strategy improves procedural efficiency while maintaining comparable safety and efficacy. Larger studies are needed to confirm these findings, as only 22 patients participated in this study, limiting statistical robustness.

## Highlights

• Primary intracardiac echocardiography (ICE) with adjunctive transesophageal echocardiography (TEE) used to assess the safety and efficacy of tricuspid procedure.
• Primary TEE with ICE used as a comparison for imaging strategy assessment.
• Primary ICE strategy showed acceptable safety and efficacy trends.
• ICE strategy linked to reduced procedure and radiation times.
• ICE strategy had similar 30-day outcomes to the TEE strategy.

## Graphical abstract

**Figure:**
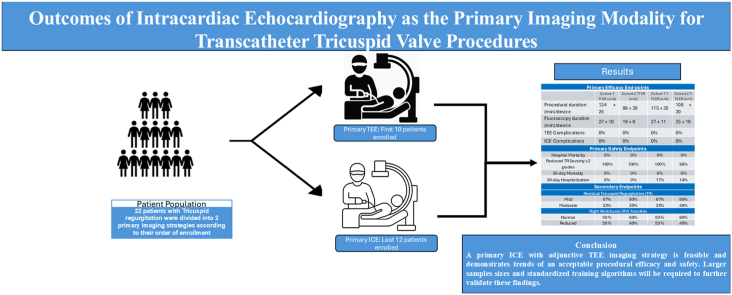

## Introduction

Tricuspid regurgitation (TR) is a condition affecting approximately 1.6 million people and carries a mortality rate of 15% to 45% depending on the patient’s comorbidities.^1^, ^2^, ^3^, ^4^ Surgical repair of the tricuspid valve carries a hospital mortality rate of 9% to 10%, though this risk decreases to 3% in specialized centers.^5^, ^6^, ^7^

Advancements in transcatheter tricuspid valve interventions, such as tricuspid transcatheter edge-to-edge repair (T-TEER) and transcatheter tricuspid valve replacement (TTVR), have shown promising outcomes. These techniques improve quality of life and reduce mortality rates to 2% to 7% at 30 days and 1 year, outperforming traditional surgical methods.^8^, ^9^, ^10^ Such procedures heavily rely on advanced transesophageal echocardiography (TEE) with two-dimensional and three-dimensional (3D) multiplanar reconstruction, which enables real-time en face valve imaging, valve and annular reconstruction, and precise positioning of the repair or replacement device.

While TEE is a powerful imaging tool to guide transcatheter procedures, TEE does have many limitations. Procedural imaging and device guidance typically require general anesthesia and do carries a significant risk for potential esophageal injury with prolonged cases.^11^ Furthermore, the anatomy and position of the tricuspid valve can lead to suboptimal visualization, as the tricuspid valve is an anterior structure, and multiple imaging artifacts and limitations can arise due to the imaging probe distance from the valve, as well as inherent limitations that arise from the esophagus, lungs, stomach, pericardium, and intracardiac devices such as pacemakers, defibrillators, and surgical rings and valves. Additional interference can come from factors such as atrial septal hypertrophy, atrial septal occlude devices, and anatomical features such as horizontal heart axis, hiatal hernias, or other potential thoracic/esophageal conditions that complicate imaging. These challenges underscore the need for adjunctive imaging modalities that circumvent these potential limitations.

Three-dimensional intracardiac echocardiography (ICE) with multiplanar reconstruction can provide a valuable adjunct by directly imaging the tricuspid valve from within the heart, thus addressing the potential inherent limitations of TEE imaging and potentially improving procedural efficiency.^12^ However, data around the safety and efficacy of ICE in tricuspid interventions are limited. As such, the aims of this study are to evaluate the use of ICE as the primary imaging tool during tricuspid valve repair and replacement procedures and to determine whether procedural outcomes and short-term patient outcomes improve as operators gain experience and proficiency with the workflow of ICE in tricuspid interventions.

## Methods

This is a single-center study conducted at the Oregon Health & Science University from November 2023 to September 2024. The study enrolled 22 consecutive patients undergoing T-TEER or TTVR. The aim was to evaluate the procedural characteristics and outcomes of 3D ICE imaging in conjunction with TEE imaging during T-TEER and TTVR procedures. Due to the differences in procedural characteristics, workflow, and device properties, TTVR and T-TEER cases were analyzed separately within each cohort.

All patients underwent baseline imaging, including transthoracic echocardiography (TTE), TEE, and cardiac computed tomography. The patients were separated into 2 cohorts. The first 10 patients made up cohort 1, in which TEE was used as the primary imaging modality for device steering, orientation, position, and deployment, while ICE was used adjunctively for confirmation. The next 12 patients made up cohort 2, in which ICE was used as the primary imaging modality for device steering, orientation, position, and deployment, while TEE was used adjunctively for confirmation. The Philips VeriSight Pro 3D ICE catheter and the EPIQ CVx Echocardiography System (Philips Healthcare, Amsterdam, the Netherlands) fit with a 3D X8-2t TEE probe were used for ICE and TEE, respectively. Final procedural outcomes were assessed using both TEE and ICE. Postprocedural echocardiographic measurements were made at 30 days using TTE to assess cardiac metrics to track improvement. TR reduction assessment was conducted on all patients to quantify the reduction in TR severity and compare outcomes across cohorts.

### Procedure Workflow

The Philips X8-2t 3D (Philips Healthcare, Amsterdam, the Netherlands) TEE probe was advanced into the esophagus and positioned to obtain high-resolution imaging of the tricuspid valve and surrounding structures (Video 1). Standard views, including the mid-esophageal four-chamber, right ventricular (RV) inflow-outflow, and bicaval views, were utilized for preprocedural and procedural assessment and guidance.^12^^,^^13^ A 10F 30 cm sheath was used to introduce the Philips VeriSight Pro (Philips, Amsterdam, the Netherlands) 3D ICE catheter into the femoral vein. The catheter was subsequently advanced to the right atrium. This approach enabled precise imaging of the tricuspid valve and surrounding structures during T-TEER and TTVR procedures (Video 2). Slight catheter manipulation was used to manage any device shadowing.^12^^,^^13^ In TTVR procedures, the EVOQUE tricuspid replacement system (Edwards Lifesciences, Irvine, CA) was utilized, while the PASCAL (Edwards Lifesciences, Irvine, CA) device was used in patients undergoing repair-TEER. The heart team performing the procedures had completed over 50 T-TEER and TTVR cases together, eliminating any learning curve associated with tricuspid device utilization.

### Data Collection

Baseline clinical data, procedural outcomes, and 30-day follow-up variables were collected retrospectively. Preprocedural TR and RV function were assessed using TTE and TEE. Additionally, baseline TR severity was quantified using the proximal isovelocity surface area method, RV function was assessed using tricuspid annular plane systolic excursion, systolic pulmonary artery pressure was determined by using the modified Bernoulli equation after obtaining the TR jet velocity with continuous wave Doppler and adding the estimated right atrial pressure, and the ejection fraction was determined using the Simpson method.^14^, ^15^, ^16^, ^17^

Procedural characteristics, including technical success, procedural time, fluoroscopy time, TEE and ICE complications, and TEE and ICE usage times, were also collected. Technical success was defined as the successful delivery of the device and the ability to visualize the anatomy sufficiently for safe device deployment. Procedural time and fluoroscopy time were recorded as averages per device, accounting for cases where multiple devices were required for repair procedures. TEE and ICE complications included perforations, bleeding events, and hematomas. TEE usage time was assessed as TEE time from procedural venous sheath insertion to removal for T-TEER and TTVR and thus did not include TEE preprocedural or TEE postprocedural imaging to ensure consistency between TEE and ICE times.

Postprocedural characteristics, including the length of hospital stay, were defined as the duration from the procedure’s completion to the patient’s final discharge home. RV function and residual TR severity were evaluated immediately after the procedure and at the 30-day follow-up using TEE and TTE, respectively.^18^^,^^19^

### Study Endpoints

Key procedural variables collected include procedural time, fluoroscopy time, residual TR, ICE/TEE complications, a decrease in residual TR by ≥ 2 grades, and 30-day mortality and hospitalizations. Additional variables include length of hospital stay and the echocardiographic parameters such as RV function and tricuspid valve function 30 days after the procedure.

### Statistical Analysis

IBM SPSS (version 30.0.0.0; IBM, Armonk, New York) was used for statistical analysis. The Mann-Whitney U test was employed to assess the differences between cohorts, given the small sample size and lack of normality. Logistic regression was used for analyzing binary outcomes and associations.

## Results

**Figure 1:**
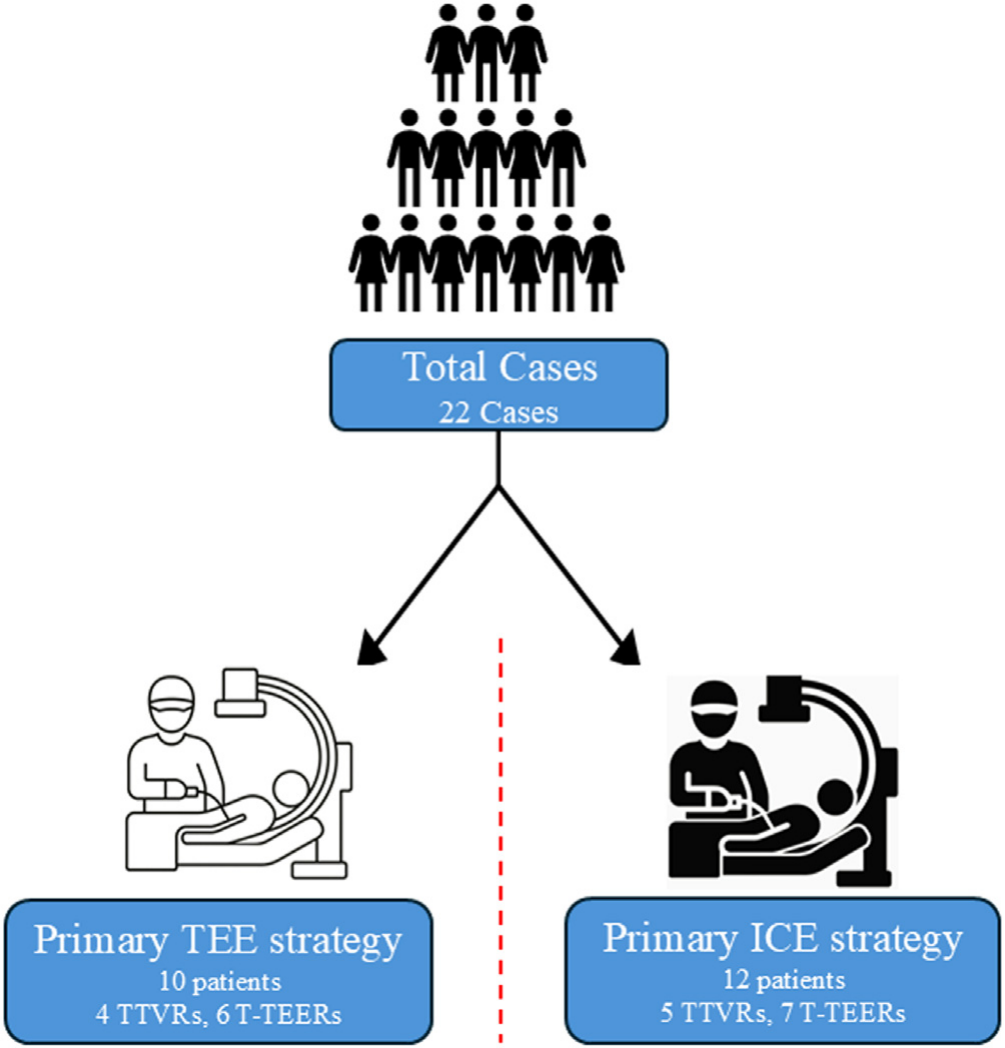
Patient stratification and procedural breakdown by imaging strategy. Flow chart illustrating the stratification of patients into 2 cohorts based on the primary imaging strategy. The chart also displays the type of procedures performed in each cohort. Abbreviations: ICE, intracardiac echocardiography; TEE, transesophageal echocardiography; T-TEER, tricuspid transcatheter edge-to-edge repair; TTVR, transcatheter tricuspid valve replacement.

The number of patients participating in this study was 22 (Figure 1). The first 10 patients were grouped into cohort 1, and the next 12 patients were grouped into cohort 2. In cohort 1, there were 10 patients, 4 receiving TTVR and 6 T-TEER devices. In cohort 2, there were 12 patients, 5 receiving TTVR and 7 T-TEER devices.

### Patient Demographic Baseline Clinical Characteristics

The patient demographics, baseline clinical labs, medical history, and surgical history were comparable between the 2 cohorts. The average age of the cohorts was approximately 60 ± 5 years, and nearly 75% had New York Heart Association (NYHA) class 3 to 4 symptoms pretreatment. Functional status by Kansas City Cardiomyopathy Questionnaire as well as renal function tended to be better in those receiving T-TEER therapies. Additionally, there was a trend toward a lower percentage of atrial arrhythmias in those receiving T-TEER as well as a trend toward reduced RV systolic function. See Table 1 for cohort 1 and cohort 2 clinical characteristics separated by cohort and treatment received.

**Table 1:** Baseline demographics, clinical characteristics, laboratory values, risk scores, and imaging findings (n = 22)

Baseline clinical data∗				
	Cohort 1 TTVR (n = 4)	Cohort 2 TTVR (n = 5)	Cohort 1 T-TEER (n = 6)	Cohort 2 T-TEER (n = 7)
Age (y)	60 ± 5	62 ± 7	58 ± 6	61 ± 4
Male gender	50%	60%	70%	55%
BMI (kg/m^2^)	28 ± 3	27 ± 2	26 ± 4	29 ± 2
NYHA functional class III or IV	75%	80%	67%	86%
KCCQ	39 ± 19	36 ± 15	46 ± 12	39 ± 10
Baseline creatinine (mg/dL)	1.01 ± 0.36	1.24 ± 0.45	0.88 ± 0.23	1.12 ± 0.43
Baseline hemoglobin (g/dL)	12.41 ± 1.69	12.38 ± 1.89	12.62 ± 1.12	12.37 ± 1.18
Comorbidities				
History of CAD	40%	30%	50%	40%
History of myocardial infarction	20%	25%	17%	43%
History of atrial fibrillation/flutter	100%	100%	83%	100%
History of diabetes	0%	20%	0%	29%
History of dyslipidemia	75%	80%	83%	86%
History of hypertension	100%	100%	100%	86%
History of stroke	0%	20%	33%	29%
History of ventricular tachycardia/fibrillation	0%	0%	0%	14%
Use of anticoagulation	100%	100%	67%	71%
History of permanent pacemaker	50%	20%	0%	29%
History of mitral valve repair	30%	30%	40%	30%
History of CABG	0%	20%	17%	0%
History of aortic valve replacement	50%	30%	60%	40%
History of pulmonary hypertension† (sPAP ≥30 mmHg)	75%	40%	67%	71%
History of renal insufficiency	0%	40%	17%	29%
History of COPD	25%	40%	0%	14%
Baseline tricuspid regurgitation‡				
Severe	75%	100%	100%	100%
Massive	25%	0%	0%	0%
Baseline right ventricular function§				
Normal	75%	80%	50%	71%
Reduced	25%	20%	50%	29%
Baseline ejection fraction‖				
Normal	100%	67%	100%	86%
Reduced	0%	33%	0%	14%

Abbreviations: BMI, body mass index; CABG, coronary artery bypass graft; CAD, coronary artery disease; COPD, chronic obstructive pulmonary disease; KCCQ, Kansas City Cardiomyopathy Questionnaire; NYHA, New York Heart Association; sPAP, systolic pulmonary artery pressure; T-TEER, tricuspid transcatheter edge-to-edge repair; TTVR, transcatheter tricuspid valve replacement.

∗ Plus–minus values are means ± SD.

† Pulmonary hypertension was evaluated by determining the tricuspid regurgitation jet velocity using color Doppler, which was used to estimate the sPAP.

‡ Tricuspid regurgitation was evaluated using the proximal isovelocity surface area method.

§ Right ventricular function was assessed using the tricuspid annular plane systolic excursion method.

‖ Ejection fraction was determined using the Simpson method.

### Procedural Characteristics

All TTVR and T-TEER procedures were deemed successful regardless of the imaging cohort. Notably, procedural time as well as fluoroscopy time for both TTVR and T-TEER treatment were reduced in cohort 2 compared to cohort 1, demonstrating time and radiation safety improvements when utilizing a primary ICE imaging strategy with TEE confirmation. For TTVR therapies, there was a reduction in the TEE procedural imaging time between cohort 1 and cohort 2, 49 ± 17 minutes vs. 12 ± 20 minutes, respectively, with a minimal change in the ICE imaging time, 30 ± 18 minutes up to 35 ± 11 minutes. For T-TEER therapies, ICE imaging time increased from 38 ± 15 minutes to 50 ± 23 minutes with similar TEE procedural imaging time. See Table 2. In total, 19 T-TEER devices were deployed across 13 procedures, with 5 patients receiving 2 clips and 1 patient receiving 3 clips. No procedural, ICE complications, or TEE complications occurred in either cohort regardless of treatment therapy.

**Table 2:** Intraprocedural characteristics, including duration metrics and device-related complications (n = 22)

Procedure characteristics∗				
	Cohort 1 TTVR (n = 4)	Cohort 2 TTVR (n = 5)	Cohort 1 T-TEER (n = 6)	Cohort 2 T-TEER (n = 7)
Technical success†	100%	100%	100%	100%
Procedural duration (min)/device‡	124 ± 25	98 ± 36	115 ± 35	108 ± 30
Fluoroscopy duration (min)/device‡	27 ± 10	19 ± 9	27 ± 11	25 ± 10
TEE complications	0%	0%	0%	0%
ICE complications	0%	0%	0%	0%
TEE time (min)§	49 ± 17	23 ± 20	43 ± 14	44 ± 28
ICE time (min)§	30 ± 18	35 ± 11	38 ± 15	50 ± 23

Abbreviations: ICE, intracardiac echocardiography; TEE, transesophageal echocardiography; T-TEER, tricuspid transcatheter edge-to-edge repair; TTVR, transcatheter tricuspid valve replacement.

∗ Plus–minus values are means ± SD.

† Technical success is defined as the ability to deliver the device successfully and the ability to see the anatomy for safe deployment of the device.

‡ Procedure and fluoroscopy times were divided by the number of clips deployed during T-TEER procedures.

§ TEE and ICE assessments before device introduction to the body and after device release were excluded from the analysis, as TEE and ICE times were strictly limited to procedural use to ensure consistency.

### Procedural Outcomes and 30-Day Follow-Up

Procedural outcomes as well as 30-day outcomes were similar between cohort 1 and cohort 2 for both TTVR and T-TEER treatments. See Table 3. There was no in-hospital or 30-day mortality for either group. While NYHA functional classification and Kansas City Cardiomyopathy Questionnaire were lower for cohort 2 than cohort 1 for each of the TR treatment options (TTVR and T-TEER), there was no statistically significant difference between the cohorts. Overall, NYHA functional class improved after treatment with 60% of subjects in NYHA class 1-2 postprocedure. There was a marked improvement in TR grade reduction across the cohorts with a ≥ 2 grade TR reduction in all but 1 patient. As expected, residual TR and 30-day heart failure hospitalizations were higher in the T-TEER group vs. TTVR group, though no intercohort difference was noted based on imaging protocol.

**Table 3:** Procedural outcomes and 30-day follow-up, including mortality, morbidity, follow-up scores, and imaging findings (n = 22)

Procedure outcomes and 30-d follow-up∗				
	Cohort 1 TTVR (n = 4)	Cohort 2 TTVR (n = 5)	Cohort 1 T-TEER (n = 6)	Cohort 2 T-TEER (n = 7)
Length of stay (d)†	4 ± 1	4 ± 1	3 ± 1	4 ± 1
Hospital mortality	0%	0%	0%	0%
Reduced TR severity ≥2 grades	100%	100%	100%	86%
NYHA functional class I or II	75%	40%	83%	57%
KCCQ	52 ± 9	42 ± 9	51 ± 13	43 ± 16
30-d mortality	0%	0%	0%	0%
30-d hospitalization	0%	0%	17%	14%
Residual TR				
Mild	67%	80%	67%	60%
Moderate	33%	20%	33%	40%
Right ventricular function				
Normal	50%	60%	50%	60%
Reduced	50%	40%	50%	40%
Ejection fraction				
Normal	100%	100%	83%	71%
Reduced	0%	0%	17%	29%

Abbreviations: KCCQ, Kansas City Cardiomyopathy Questionnaire; NYHA, New York Heart Association; TR, tricuspid regurgitation; T-TEER, tricuspid transcatheter edge-to-edge repair; TTVR, transcatheter tricuspid valve replacement.

∗ Plus–minus values are means ± SD.

† Length of stay starts from the time of procedure completion and excludes preprocedural admission.

## Discussion

This single-center study evaluated the impact of a primary ICE-guided imaging modality with TEE confirmation in consecutive patients undergoing TTVR or T-TEER. The findings demonstrated the following: 1) Procedural time for both TTVR and T-TEER treatments was reduced when using a primary ICE-guided imaging strategy with adjunctive TEE. 2) Fluoroscopy time was similarly reduced in primary ICE-guided procedures. 3) There were no differences in patient safety or 30-day outcomes when using a primary ICE with adjunctive TEE strategy for TTVR and T-TEER therapies.

The use of ICE has been described in a wide range of structural heart procedures, including tricuspid and mitral valve repair or replacement, left atrial appendage occlusion, paravalvular leak repair, transeptal puncture, patent foramen ovale closure, and transcatheter pulmonary valve replacement.^9^ While ICE offers clear advantages in right-sided procedures, TEE is often preferred for left-sided interventions due to the esophagus’s proximity to the left atrium. ICE, however, can also be successfully utilized in left-sided procedures such as mitral TEER, transcatheter mitral valve replacement, and left atrial appendage occlusion, demonstrating its expanding versatility. Additionally, ICE has the potential to enhance transcatheter pulmonary valve replacement procedures, which currently rely heavily on fluoroscopy and TEE.

Two-dimensional ICE provides high spatial and temporal resolution, with frame rates comparable to or exceeding those of two-dimensional TEE. Similarly, 3D ICE and 3D TEE offer nearly equivalent frame rates. Given this, ICE and TEE offer comparable image quality, and the choice between them should be based on procedural anatomy and access considerations.

The results of this study align with findings from pivotal trials such as von Bardeleben et al.,^20^ Zahr et al.,^21^ and Arnold et al.^22^ In von Bardeleben et al.,^20^ the mean procedural time was 90 ± 66 minutes, comparable to the times observed in the ICE-guided cohorts of this study. Zahr et al.^21^ demonstrated a ≥2-grade TR reduction in 75% of patients, aligning with the outcomes of this study, where nearly all patients achieved this endpoint. Unlike prior studies, which primarily relied on TEE or used ICE adjunctively, this study uniquely evaluated whether a TEE with adjunctive ICE (cohort 1) compared to primary ICE with adjunctive TEE (cohort 2) led to any significant differences in procedure success, procedural times, as well as short-term and 30-day outcomes, allowing for a direct comparison of the 2 strategies. Trends toward reduced procedural time and fluoroscopic exposure in ICE-guided procedures were evident, underscoring the potential of ICE as a safer and more efficient alternative. In contrast to Wang et al.,^23^ who observed no significant differences in procedural or fluoroscopic times when ICE was used adjunctively, this study highlights the reduction in procedural and fluoroscopic times when ICE is used as the primary imaging modality in tricuspid valve interventions indicating the potential of ICE to be used as primary imaging modality for tricuspid interventions.

### Limitations

This study has many limitations. First, the study contained a relatively small sample size, and statistical significance was not achieved. Second, the study was completed in a single center without core lab adjudication. Third, the study’s design limits the ability to draw conclusions about the superiority of 1 imaging modality over the other. Despite the fact that the operators had prior experience with the tricuspid intervention devices, the learning curve of ICE was not accounted for.

## Conclusion

A primary ICE with adjunctive TEE imaging strategy demonstrates an improved procedural efficacy in the reduction of procedure and fluoroscopy times while maintaining comparable benefits in the treatment of TR compared to a standard primary TEE with adjunctive ICE strategy. Moreover, the primary ICE-guided TTVRs and T-TEERs showed similar safety when compared to the standard TEE-guided approach, with noted similar hospitalization, mortality, and major bleeding events across both cohorts. Larger sample sizes are needed to further validate these findings, as the current study included only 22 patients, limiting statistical robustness and the ability to draw definitive conclusions. Additionally, standardized training protocols will be essential to evaluate the efficacy and safety of ICE as the primary imaging modality for treating severe TR. While the results are not conclusive, the observed trends provide a foundation for hypothesis generation and underscore the need for larger, more definitive studies in this area.

## Review Statement

The review of this paper was handled by Guest Editor Mani Vannan, MD.

## Ethics Statement

This study was conducted in accordance with the Declaration of Helsinki and its subsequent amendments, Good Clinical Practice principles, and ISO 14155. The protocol was approved by the Oregon Health & Science University Institutional Review Board or Ethics Committee.

## Funding

This study was partially funded by Philips Healthcare. The sponsor was not involved in study design, data collection, analysis, interpretation, manuscript writing or submission. All images and videos are original and were created by the authors.

## Disclosure Statement

F. E. Zahr consults for and is a recipient of educational and research grants from Edwards, Medtronic, Philips, and GE. S. M. Chadderdon consults for and is a recipient of educational and research grants from Edwards, Medtronic, Philips, and GE.

## Supplementary Material

Video 1 Two-dimensional transgastric TEE with color Doppler showing successful device deployment on septal and anterolateral leaflets. Mild residual TR can be seen posterior to the implant. Abbreviations: TEE, transesophageal echocardiography; TR, tricuspid regurgitation.

Video 2 Two-dimensional ICE with color Doppler confirming successful T-TEER device deployment on septal and anterolateral leaflets. Abbreviations: ICE, intracardiac echocardiography; T-TEER, tricuspid transcatheter edge-to-edge repair.

## References

[1] Fender E.A., Zack C.J., Nishimura R.A. (2018). Isolated tricuspid regurgitation: outcomes and therapeutic interventions. Heart.

[2] Topilsky Y., Maltais S., Medina Inojosa J. (2019). Burden of tricuspid regurgitation in patients diagnosed in the community setting. JACC Cardiovasc Imaging.

[3] Nath J., Foster E., Heidenreich P.A. (2004). Impact of tricuspid regurgitation on long-term survival. J Am Coll Cardiol.

[4] Benfari G., Antoine C., Miller W.L. (2019). Excess mortality associated with functional tricuspid regurgitation complicating heart failure with reduced ejection fraction. Circulation.

[5] Zack C.J., Fender E.A., Chandrashekar P. (2017). National trends and outcomes in isolated tricuspid valve surgery. J Am Coll Cardiol.

[6] Kilic A., Saha-Chaudhuri P., Rankin J.S., Conte J.V. (2013). Trends and outcomes of tricuspid valve surgery in North America: an analysis of more than 50,000 patients from the Society of Thoracic Surgeons database. Ann Thorac Surg.

[7] Hamandi M., Smith R.L., Ryan W.H. (2019). Outcomes of isolated tricuspid valve surgery have improved in the modern era. Ann Thorac Surg.

[8] Kodali S.K., Hahn R.T., Davidson C.J. (2023). 1-Year outcomes of transcatheter tricuspid valve repair. J Am Coll Cardiol.

[9] Wang X., Ma Y., Liu Z. (2023). Comparison of outcomes between transcatheter tricuspid valve repair and surgical tricuspid valve replacement or repair in patients with tricuspid insufficiency. J Cardiothorac Surg.

[10] Shimoda T.M., Ueyama H.A., Miyamoto Y. (2025). Comparison of transcatheter versus surgical tricuspid repair among patients with tricuspid regurgitation: two-year results. Circ Cardiovasc Interv.

[11] Freitas-Ferraz A.B., Bernier M., Vaillancourt R. (2020). Safety of transesophageal echocardiography to guide structural cardiac interventions. J Am Coll Cardiol.

[12] Tang G.H.L., Zaid S., Hahn R.T. (2025). Structural heart imaging using 3-dimensional intracardiac echocardiography: JACC: cardiovascular imaging position statement. JACC Cardiovasc Imaging.

[13] Chadderdon S.M., Eleid M.F., Thaden J.J. (2022). Three-dimensional intracardiac echocardiography for tricuspid transcatheter edge-to-edge repair. Struct Heart.

[14] Zoghbi W.A., Adams D., Bonow R.O. (2017). Recommendations for Noninvasive evaluation of Native valvular regurgitation: a report from the American Society of Echocardiography developed in collaboration with the Society for Cardiovascular Magnetic Resonance. J Am Soc Echocardiogr.

[15] Rudski L.G., Lai W.W., Afilalo J. (2010). Guidelines for the echocardiographic assessment of the right heart in adults: a report from the American Society of Echocardiography endorsed by the European Association of Echocardiography, a registered branch of the European Society of Cardiology, and the Canadian Society of Echocardiography. J Am Soc Echocardiogr.

[16] Lang R.M., Badano L.P., Mor-Avi V. (2015). Recommendations for cardiac chamber quantification by echocardiography in adults: an update from the American Society of Echocardiography and the European Association of Cardiovascular Imaging. J Am Soc Echocardiogr.

[17] Nagueh S.F., Smiseth O.A., Appleton C.P. (2016). Recommendations for the evaluation of left ventricular diastolic function by echocardiography: an update from the American Society of Echocardiography and the European Association of Cardiovascular Imaging. J Am Soc Echocardiogr.

[18] (2011). NEW EAE/ASE recommendations for the use of echocardiography in new transcatheter interventions for valvular heart disease. Eur Heart J.

[19] Hahn R.T., Abraham T., Adams M.S. (2013). Guidelines for performing a comprehensive transesophageal echocardiographic examination: recommendations from the American Society of Echocardiography and the Society of Cardiovascular Anesthesiologists. J Am Soc Echocardiogr.

[20] von Bardeleben R.S., Lurz P., Sorajja P. (2023). Two-year outcomes for tricuspid repair with a transcatheter edge-to-edge valve repair from the transatlantic TRILUMINATE trial. Circ Cardiovasc Interv.

[21] Zahr F., Smith R.L., Gillam L.D. (2023). One-year outcomes from the CLASP IID randomized trial for degenerative mitral regurgitation. JACC Cardiovasc Interv.

[22] Arnold S.V., Hahn R.T., Thourani V.H. (2025). Quality of life after transcatheter tricuspid valve replacement: 1-year results from TRISCEND II pivotal trial. J Am Coll Cardiol.

[23] Wang L., Petrossian G., Robinson N. (2024). Early experience of 3-dimensional intracardiac echocardiography in transcatheter tricuspid interventions. J Soc Cardiovasc Angiogr Interv.

